# Quantitation of Exosomes and Their MicroRNA Cargos in Frozen Human Milk

**DOI:** 10.1097/PG9.0000000000000172

**Published:** 2022-02-04

**Authors:** Haichuan Wang, Di Wu, Sonal Sukreet, Anthony Delaney, Mandy B. Belfort, Janos Zempleni

**Affiliations:** From the * Department of Nutrition and Health Sciences, University of Nebraska-Lincoln; † Department of Pediatric Newborn Medicine, Brigham and Women’s Hospital, Boston, MA.

**Keywords:** authentication, detection, epidemiology, infants, stability

## Abstract

We assessed feasibility of analyzing exosomes and microRNA cargos in frozen human milk as a prerequisite for epidemiological studies of milk exosomes. We collected milk from 5 mother-preterm infant dyads at 3 time points during postnatal hospital care for storage at −80 °C. We purified exosomes by ultracentrifugation, probed marker proteins using immunoblots, assessed size and counts with a nanoparticle tracker, and quantified 3 microRNAs with quantitative PCR. Positive exosome marker proteins were detectable; β-casein was the only detectable contaminant. Exosome count and size trended to decrease from early to late samples (count, 2.3 × 10^9^ ± 3.8 × 10^9^ to 5.6 × 10^8^ ± 9.7 × 10^8^ exosomes/mL; size, 117 ± 25 to 92 ± 16 nm). Two microRNAs were detectable in early samples only; cycle threshold values equaled 28.7 ± 0.7 for miR-30d-5p and miR-125a-5p; miR-423-5p was not detectable. We conclude that the analysis of exosomes and quantification of microRNAs is feasible in human milk previously stored at −80 °C.

**What Is Known**

- Exosomes play an important role in cell-to-cell communication.
- Milk exosomes and their microRNA cargos are bioavailable.
- Dietary depletion of milk exosomes and their microRNA cargos elicits phenotypes in infants and piglets.

**What Is New**

- Human milk may be frozen at −80 °C for subsequent analysis of exosomes and microRNAs.
- Interindividual variability is large for exosomes and their microRNA cargos in frozen human milk.
- Studies of human milk exosomes are feasible in large cohorts of mother-infant pairs.

## INTRODUCTION

Exosomes are increasingly implicated in child health and disease across numerous organ systems, including the gastrointestinal system [1]. Exosomes facilitate cell-to-cell communication by binding to receptors on the surface of recipient cells and transferring their cargos from donor cells to recipient cells [2]. MicroRNA cargos are of particular importance because they regulate the expression of more than 60% of human genes; loss of microRNA biogenesis is embryonic lethal [3,4]. Human milk contains large amounts of exosomes, which encapsulate microRNAs and thereby protect them against degradation by low pH and RNases within the gastrointestinal tract [5]. Exosomes and microRNAs are absorbed from milk and contribute to plasma and tissue microRNA content [6–8]. Dietary depletion of milk exosomes and their microRNA cargos leads to an increased severity of inflammation in a mouse model of inflammatory bowel disease and a decrease in postnatal survival to weaning in mice [7,8]. Milk exosomes increased villi height and crypt depth in the duodenum, prevented the development of necrotizing enterocolitis, and helped to maintain intestinal epithelial barrier integrity in mice [9,10]. Because of these previous observations it is important to lay the groundwork for large epidemiological studies that compare the health and well-being of breastfed and formula-fed infants; infant formulas are essentially free of exosomes and microRNAs [11].

Milk exosomes have been identified as a research priority in the 2020–2030 Strategic Plan for National Institutes of Health Nutrition Research [12]. Epidemiological studies of milk exosomes in large cohorts require that milk be frozen upon collection and, therefore, require the demonstration that exosomes and their microRNA cargos are detectable in frozen samples. Exosome purification from fresh milk at a clinical or community-based research site is not feasible because it requires equipment such as ultracentrifuges, high-speed centrifuges, affinity chromatography, or size exclusion chromatography [13]. A previous study reported a loss of exosome-sized vesicles in milk during storage at −80 °C, but exosomes were identified without prior purification and purely based on size [11]. Likely, particles of sizes similar to exosomes were included in the count, eg, fat globules, casein micelles, and small microvesicles (See Supplemental Digital Content Table 1, http://links.lww.com/PG9/A68). The objectives of this study were to (1) determine whether exosomes and their microRNA cargos can be quantified in small volumes of frozen human milk, thereby facilitating epidemiological studies of possible health benefits of milk exosomes in infants and (2) compare exosomal microRNA cargos in milk collected in early, mid, and late stages of hospitalization.

## METHODS

### Participants

We leveraged banked, frozen human milk specimens that were previously collected for an observational study of human milk macronutrient content and outcomes in very preterm (<32 weeks of gestation) infants [14]. Briefly, mothers expressed milk for their hospitalized infants, and nurses collected 3 to 5 mL milk samples at the bedside just prior to feeding the infant via gavage tube (See Supplemental Digital Content Figure 1, http://links.lww.com/PG9/A71). Milk may have been pooled from >1 expression and may have included fresh or previously frozen milk. This approach ensured that milk samples represented the actual infant diets. After collection, milk was refrigerated at 4 °C for up to 12 hours in the clinical setting and then frozen at −80 °C for up to 6 months. For this study, we selected 5 mother-infant dyads with available samples early (range, 8–15 days), near the midpoint (range, 17–32 days), and late (range, 33–49 days) in the infant’s hospitalization. Hospitalization is the equivalent of infant age. Samples were thawed at 23 °C, aliquoted, refrozen at −80 °C, and shipped on dry ice from Boston, MA to Lincoln, NE. The study was approved by the Partners Human Research Committee (FWA00000484), and parents provided written informed consent for infants to participate.

### Exosome Isolation and Authentication

Milk samples were thawed at 23 °C, and exosomes were isolated from 1 mL human milk by using differential ultracentrifugation as previously described with minor modifications [11]. Experimental details have been deposited in extracellular vesicle (EV)-Track (https://evtrack.org) and can be accessed through EV-Track ID: EV210145. Protocols used are consistent with recommendations by the International Society for Extracellular Vesicles and the National Institutes of Health exRNA Consortium [13,15]. Exosomes were resuspended in 200 μL PBS, and counts and sizes were assessed using a NanoSight NS300 instrument. Exosome proteins were extracted using ice-cold radioimmunoprecipitation assay buffer (Sigma-Aldrich) with protease inhibitor cocktail (Sigma-Aldrich). Antibodies against CD9 (GeneTex, Irvine, CA), CD63 (Santa Cruz, Dallas, TX), and Alix (Santa Cruz) were used as positive controls; antibodies against α-tubulin (Santa Cruz) and integrin-β (Abcam, Cambridge, MA) were used to assess contamination with microvesicles. We probed human caseins in EV preparations from human milk with anti-human β-casein (Novus Biologicals, Littleton, CO). Anti-α-lactalbumin (Abcam) and anti-butyrophilin (R&D Systems, Minneapolis, MN) were used to probe whey proteins and lipid globules, respectively.

### MicroRNA Analysis

RNA was purified from exosomes (from 1 mL milk) using the miRNeasye MinElute spin, columns were treated with sodium hypochlorite to remove possible contamination, and cDNA was synthesized by using the miScript II RT kit following the manufacturer’s recommendations (Qiagen, Germantown, MD). Efficiency of RNA extraction and quantitative real-time polymerase chain reaction (qPCR), a non-natural microRNA (miSpike; IDT DNA, Coralville, IA; 5′-rCrUrCrArGrGrArUrGrGrCrGrGrArGrCrGrGrUrCrU-3′), was used in experiment as described by Wang et al [16]. qPCR was used to assess the expression of miR-30d-5p, miR-125a-5p, and miR-423-5p, which are among the 10 most abundant microRNAs in human milk exosomes [11]. qPCR was conducted using the miScript SYBR green PCR kit (Qiagen) and PCR primers as described previously [11]. PCR reactions yielded a single product judged by melting curve analysis (See Supplemental Digital Content Figure 2, http://links.lww.com/PG9/A72). Cycle threshold values greater than 29 were considered not detectable [16].

### Statistical Analyses

Exosome counts and sizes from early, mid, and late stages of hospitalization were analyzed using 1-way ANOVA. Dunnett’s post hoc test was used to compare data from mid and late hospitalization to that of early hospitalization. IBM SPSS Statistics (version 27) was used for analyses. Data are expressed as mean ± SD. Differences were considered significant if *P* < 0.05.

## RESULTS

### Exosome Content and Size

**FIGURE 1.**
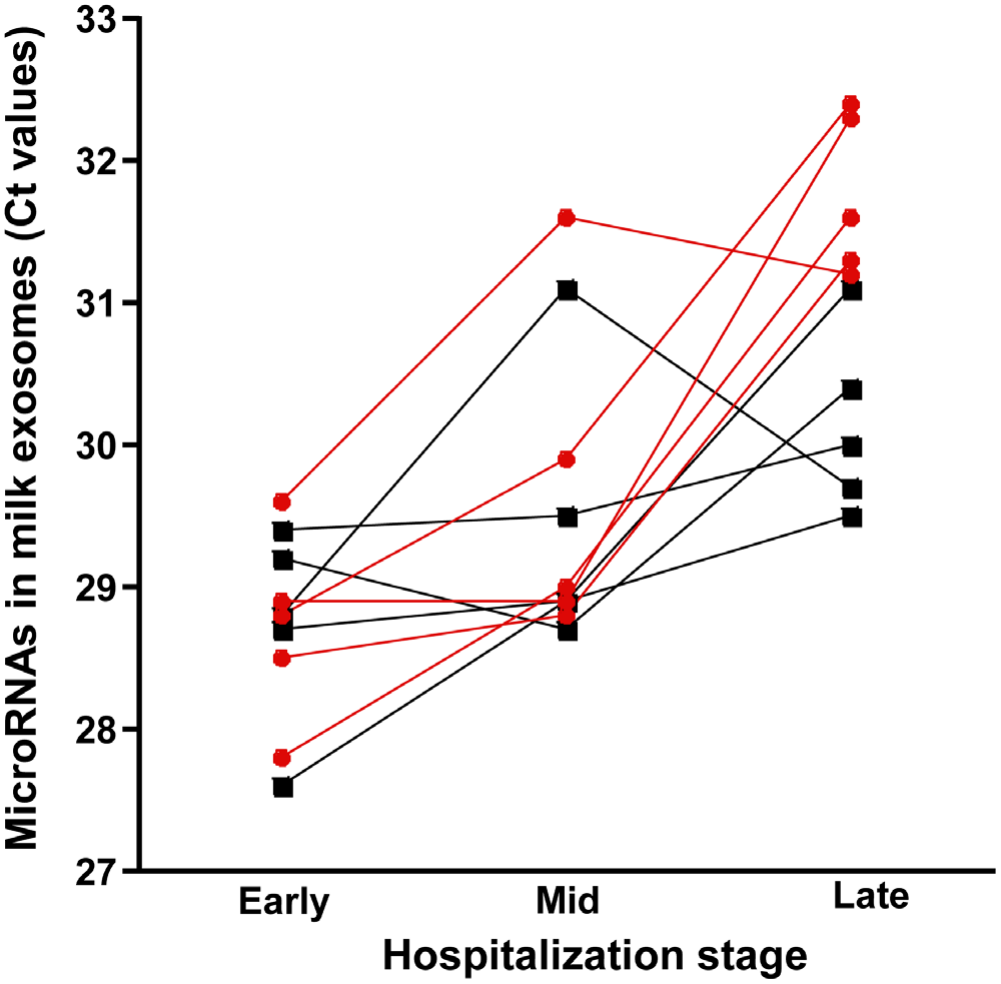
Change in cycle threshold (Ct) values of miR-30d-5p and miR-125a-5p in previously frozen milk from 5 mother-preterm infant dyads during hospitalization. Each participant is represented by a different line. miR-30d-5p is shown in black and squares; miR125a-5p is shown in red and circles.

**FIGURE 2.**
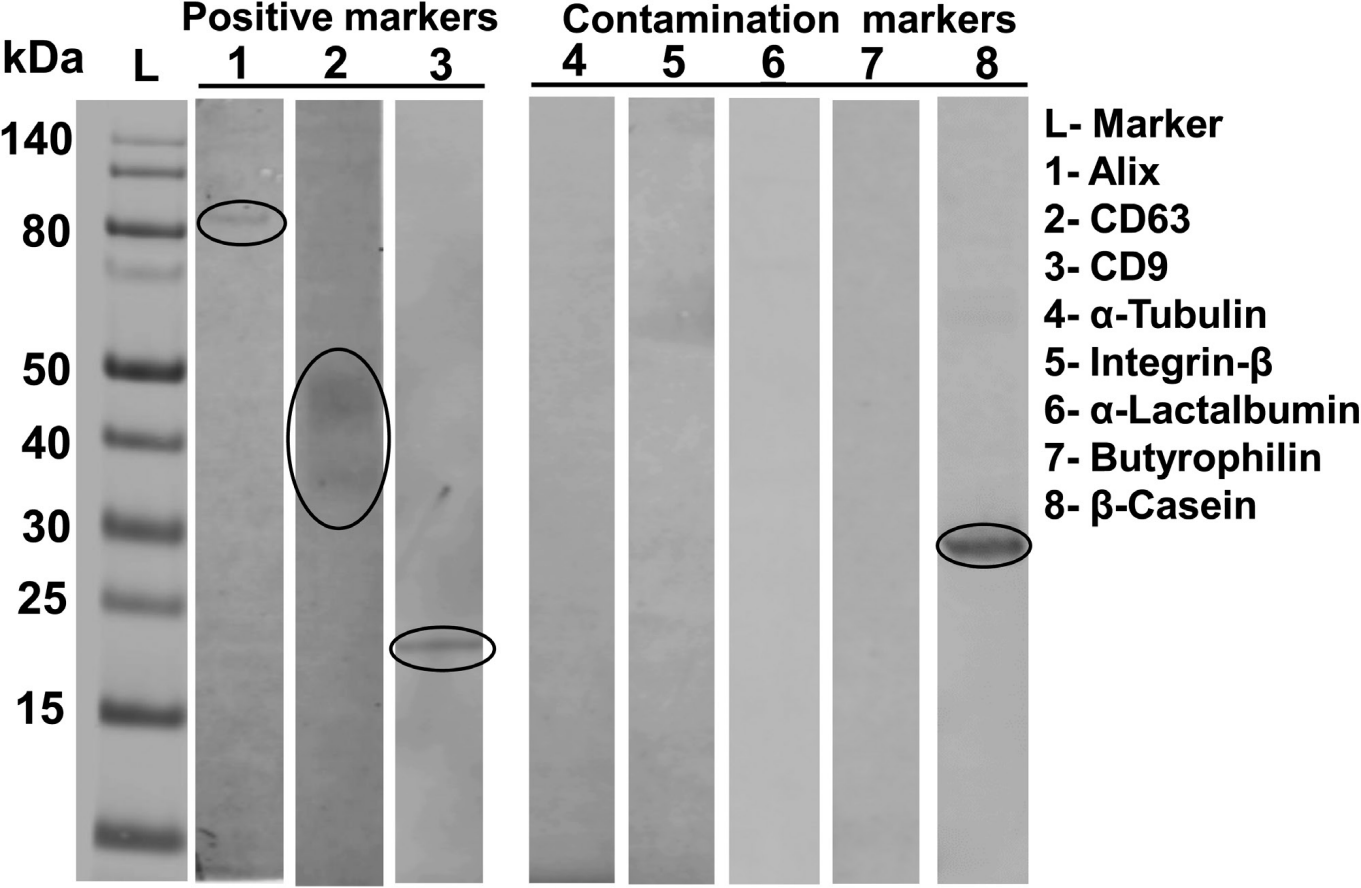
Marker proteins in HME preparations. Forty micrograms proteins were loaded per lane. HME = human milk exosomes.

Both exosome count and size decreased progressively during stages of hospitalization (Fig. 1; See Supplemental Digital Content Table 2, http://links.lww.com/PG9/A69), although differences were not statistically significant (See Supplemental Digital Content Table 3, http://links.lww.com/PG9/A70). We detected positive exosome markers, CD9, CD63, and Alix, in our preparations, whereas markers for fat globules (butyrophilin), whey proteins (α-lactalbumin), and small microvesicles (α-tubulin and integrin-β) were below detection limit. Human β-casein was present in samples (Fig. 2).

### MicroRNAs in Exosomes

Both miR-30d-5p and miR-125a-5p were detectable in exosomes from milk collected early in hospitalization and decreased to below detection limit in mid and late hospitalization (See Supplemental Digital Content Table 1, http://links.lww.com/PG9/A68). miR-423-5p was not detectable in any stage.

## DISCUSSION

Human milk has well-documented health benefits for infants, including unique benefits for preterm infants [17]. The underlying mechanisms remain unclear but are likely to involve human milk bioactives. Large cohort studies that link milk bioactives with infant health outcomes can serve as an important evidence bridge between preclinical models and eventual clinical treatment or prevention trials, [18] but a pragmatic challenge is that collecting milk from women in clinical or community sites typically does not permit for sophisticated processing or storage of samples at the point of collection. In the case of milk exosomes, previous human studies have relied on small numbers of fresh samples collected from a convenience sample of lactating women [11]. Here we demonstrate the feasibility of isolating exosomes from human milk samples that were previously collected and stored at −80 °C and the feasibility of quantifying microRNAs in frozen samples collected in the early hospitalization days 8 to 15. We further generated first insights in the large interindividual variation in exosome counts in human milk, and we provided preliminary evidence that the exosome count may decrease in late stages of hospitalization in premature infants.

Strengths of this study include the extensive use of exosome authentication protocols, as well as a qPCR protocol that has been validated in previous studies [16]. As expected, 3 positive exosome markers (CD9, CD63, and Alix) were at detectable limit, even though a trace of human β-casein was also detected in our exosome samples, which we also observed in a previous study [11]. The traces of casein are caused by incomplete separation of exosomes and casein micelles by ultracentrifugation of milk. The cycle threshold values reported here for microRNAs are similar to cycle threshold values in a previous study of exosome-sized vesicles from human milk [11]. In contrast, the exosome counts reported in this article are approximately two orders of magnitude lower than that of exosome-sized vesicles in fresh human milk from mothers giving birth to term infants (T. Kerr et al, unpublished, and Leiferman et al[11]). We are currently assessing the storage stability of purified exosomes and exosomes frozen in the milk matrix. We speculate that a loss of exosomes during storage caused to drop miR-30d-5p and miR-125a-5p to below detection limit in samples collected in mid and late hospitalization when the levels of these microRNAs are presumably lower than in early hospitalization.

As expected, we detected β-casein in our milk exosome preparations [19]. Theoretically, exosomes can be further enriched by using immunoaffinity capture, or β-casein can be removed by EDTA precipitation. That said, the use of immunoaffinity capture seems cost-prohibitive in large epidemiological studies due to the high cost of antibodies, and EDTA may interfere with the analysis of microRNAs by qPCR. Note that extracellular vesicles and particles other than exosomes contain little RNA, and exosomes are the only vesicle known to protect their RNA cargo [5,20].

We acknowledge that the sample size of this study is small, raising concerns that the decrease in exosome and microRNA content during storage might not be generalizable. The main purpose of this brief report is to document that microRNAs are detectable in small volumes of human milk previously frozen at −80 °C, thereby providing a rationale for future epidemiological studies, including studies that compare fresh and stored human milk. Future studies will need to explore storage at −20 °C as that is more readily available for large-scale epidemiological studies, especially if samples are being collected in the home environment. The feasibility of investigating human milk exosomes in frozen milk samples is critical for the conduct of large cohort studies that are optimally designed to detect associations of exosomes and their cargos with clinically important outcomes and ultimately to inform novel fortification and supplementation strategies.

M.B.B. was supported by the Brigham and Women’s Hospital Stork Fund, Brigham Research Institute Fund to Sustain Research Excellence, and the Harvard Clinical and Translational Science Center (National Center for Advancing Translational Science, grants 1UL1TR001102 and 1UL1TR002541-01). J.Z. receives research support from the National Institute of Food and Agriculture, US Department of Agriculture, under award numbers 2016-67001-25301 and 2020-67017-30834, National Institutes of Health grants 1P20GM104320 and R21OD028749, the University of Nebraska Agricultural Research Division (Hatch Act), US Department of Agriculture multistate group W4002. J.Z. serves as a consultant for PureTech Health, Inc.

## ACKNOWLEDGMENTS

The authors acknowledge the use of the Biomedical and Obesity Research Core in the Nebraska Center for the Prevention of Obesity Disease Through Dietary Molecules (NIH 1P20GM104320) at the University of Nebraska-Lincoln. H.W. acquired data, made substantial contributions to the interpretation of data, drafted and revised this work, gave final approval of the work to be published, and agreed to be accountable for all aspects of the work. D.W. acquired data, gave final approval of the work to be published, and agreed to be accountable for all aspects of the work. S.S. acquired data, gave final approval of the work to be published, and agreed to be accountable for all aspects of the work. A.D. acquired data, gave final approval of the work to be published, and agreed to be accountable for all aspects of the work. M.B.B. conceived the idea for this work, collected samples, made substantial contributions to the interpretation of data, drafted and revised this work, gave final approval of the work to be published, and agreed to be accountable for all aspects of the work. J.Z. conceived the idea for this work, made substantial contributions to the interpretation of data, drafted and revised this work, gave final approval of the work to be published, and agreed to be accountable for all aspects of the work.

## Supplementary Material


