# Supplementary material for: Quantitation of Exosomes and Their MicroRNA Cargos in Frozen Human Milk
Source: JPGN Rep. 2022 Feb 4;3(1):e172. doi: 10.1097/PG9.0000000000000172 (PMC9258983; doi:10.1097/PG9.0000000000000172)
Supplement: Supplementary file 1 [file pg9-3-e172-s001.pdf]

Supplemental Table 1. Sizes of exosomes, microvesicles and particles in milk

Vesicle/particle	Size range
	nm
Exosomes	30 - 120
Microvesicles	100 – 1,000
Casein micelles	50 - 600
Fat globules	100 – 15,000
