# Supplementary material for: Quantitation of Exosomes and Their MicroRNA Cargos in Frozen Human Milk
Source: JPGN Rep. 2022 Feb 4;3(1):e172. doi: 10.1097/PG9.0000000000000172 (PMC9258983; doi:10.1097/PG9.0000000000000172)
Supplement: Supplementary file 2 [file pg9-3-e172-s002.pdf]

Supplemental Table 2. Exosome count, size and microRNA expression in previously frozen human milk from distinct lactational stages (n = 5)

	Lactational stage		
**Variable**	**Early**	**Mid**	**Late**
Exosomes/mL milk	$2.0 \times 10^9 \pm 3.8 \times 10^9$	$1.9 \times 10^9 \pm 2.4 \times 10^9$	$5.6 \times 10^8 \pm 9.7 \times 10^8$
Exosome size (nm)	117 ± 25.1	105 ± 20.9	92 ± 15.5
miR-30d-5p (Ct)**	28.7 ± 0.7	N.D. (29.4 ± 1.0)	N.D. (30.1 ± 0.9)
miR-125a-5p (Ct)	28.7 ± 0.7	N.D. (29.6 ± 1.2)	N.D. (31.8 ± 0.5)
miR-423-5p (Ct)	N.D. (32.2 ± 0.7)	N. D. (31.4 ± 1.5)	N. D. (32.1 ± 0.2)

**Detection limit equals 29 PCR cycles under the conditions used here.

Abbreviations: Ct, cycle threshold; N.D., not detectable.
