# Supplementary material for: Quantitation of Exosomes and Their MicroRNA Cargos in Frozen Human Milk
Source: JPGN Rep. 2022 Feb 4;3(1):e172. doi: 10.1097/PG9.0000000000000172 (PMC9258983; doi:10.1097/PG9.0000000000000172)
Supplement: Supplementary file 3 [file pg9-3-e172-s003.pdf]

Supplemental Table S3. One-way ANOVA analysis of exosome count and size from different lactational stages (n = 5)

Dependent Variable			Mean Difference	Std. Error	*p*	95% Confidence Interval	
						Lower Bound	Upper Bound
**Exosome size**	**Late**	**Early**	-25.14	13.19	0.14	-58.15	7.87
	**Mid**	**Early**	-12.64	13.19	0.55	-45.65	20.37
**Exosome count**	**Late**	**Early**	-1.48E+09	1.67E+09	0.594	-5.66E+09	2.70E+09
	**Mid**	**Early**	-1.67E+08	1.67E+09	0.993	-4.35E+09	4.01E+09

*$P < 0.05$ vs. early lactation.
