# Supplementary figures and images for: Quantitation of Exosomes and Their MicroRNA Cargos in Frozen Human Milk
Source: JPGN Rep. 2022 Feb 4;3(1):e172. doi: 10.1097/PG9.0000000000000172 (PMC9258983; doi:10.1097/PG9.0000000000000172)

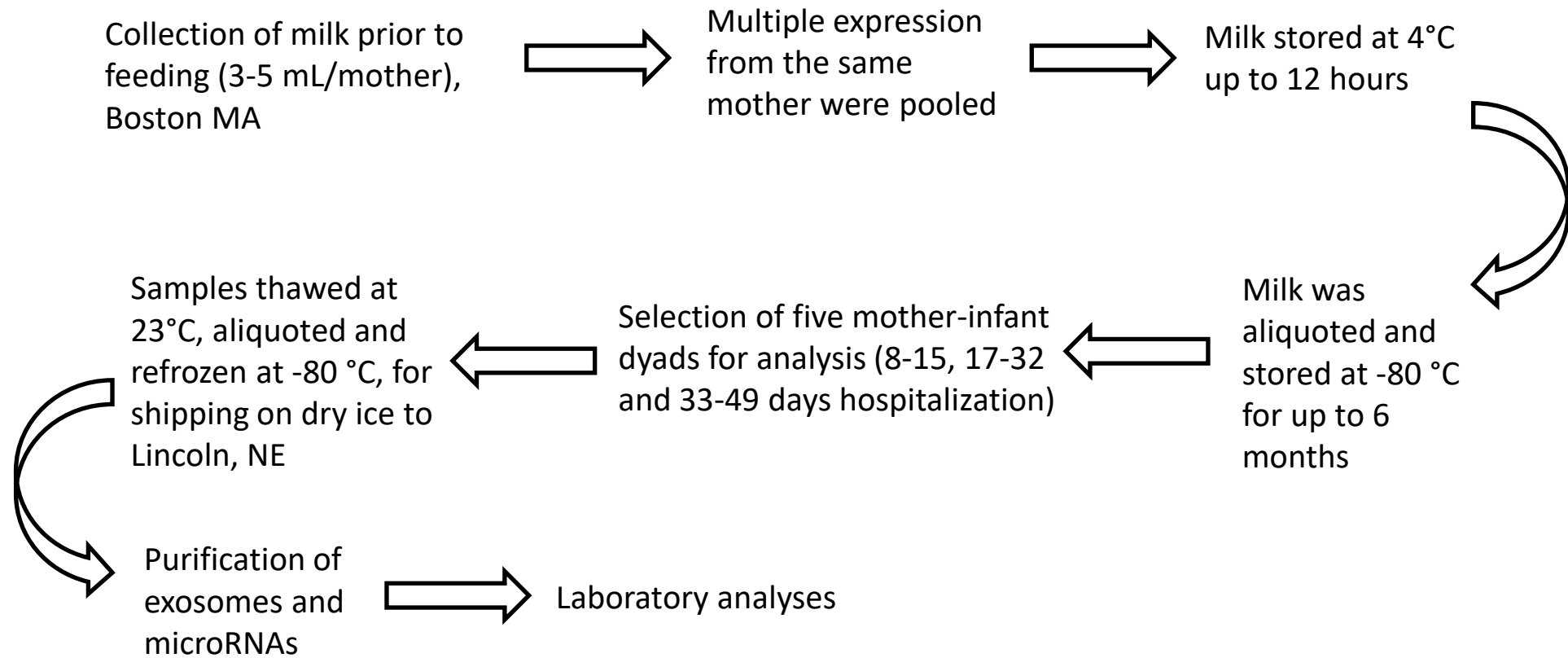

Fig. S1. Flow chart of human milk sample collection and process

Supplement: Supplementary file 4 [file pg9-3-e172-s004.pdf]

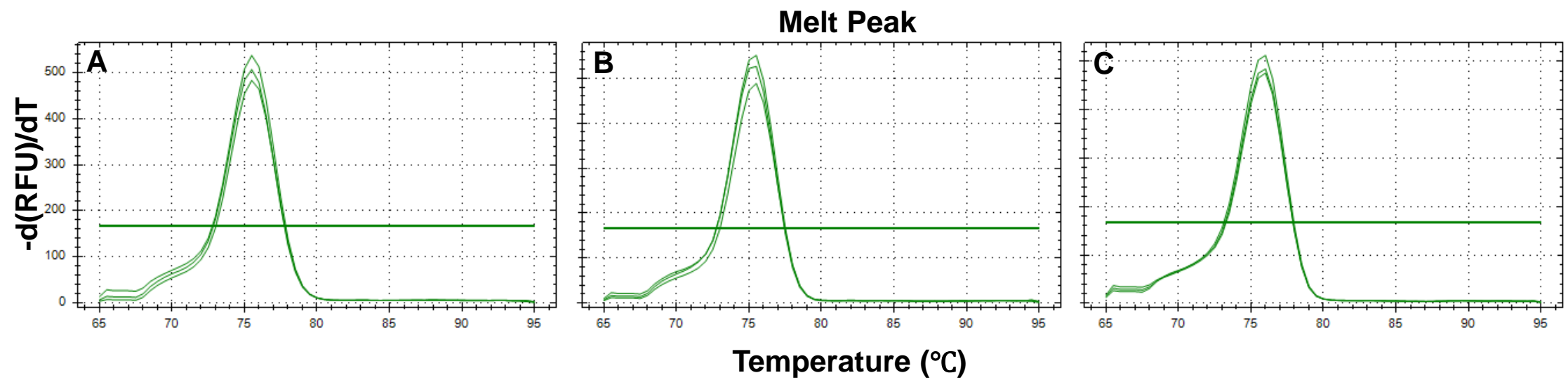

Fig. S2. Melting curves in qPCR analysis of miR-30d-5p (A), miR125a-5p (B) and miR-423-5p (C).

Supplement: Supplementary file 5 [file pg9-3-e172-s005.pdf]
